# A new species and first record of the genus *Cynegetis* Chevrolat (Coleoptera, Coccinellidae, Epilachnini) from China

**DOI:** 10.3897/zookeys.448.8342

**Published:** 2014-10-20

**Authors:** Xingmin Wang, Wioletta Tomaszewska, Shunxiang Ren

**Affiliations:** 1 Engineering Research Center of Biological Control, Ministry of Education, South China Agricultural University, Guangzhou, 510642 China; 2 Museum and Institute of Zoology, Polish Academy of Sciences, 00–679 Warszawa, Poland

**Keywords:** Entomology, taxonomy, Cucujoidea, *Cynegetis*, new species, China

## Abstract

The first species of the genus *Cynegetis* Chevrolat is recorded from China. *Cynegetis chinensis* Wang & Ren, **sp. n.** is described from the Ningxia Province in North China. A key to the known species of *Cynegetis* is given. Diagnostic similarities and differences between *Cynegetis* and *Subcoccinella* Agassiz & Erichson are discussed and illustrated.

## Introduction

The genus *Cynegetis* was established by Chevrolat (Chevrolat in Dejean 1837) and the type species, *Cynegetis impunctata* (Linnaeus, 1767) was designed by Crotch in 1874. Formerly *Cynegetis* was classified in the tribe Cynegetini Thomson (= Madaini Gordon) in the subfamily Epilachninae.

The subfamily Epilachninae traditionally was divided into four tribes: Epilachnini Mulsant, Madaini Gordon, Epivertini Pang & Mao and Eremochilini Gordon & Vandenberg (Jadwiszczak and Węgrzynowicz 2003), but recently Seago et al. (2011) combined these into a single tribe Epilachnini within a broadly defined subfamily Coccinellinae. As currently defined, Epilachnini is a large group of herbivorous ladybird beetles that include 25 genera with a worldwide distribution (Jadwiszczak and Węgrzynowicz 2003, Szawaryn 2011, Szawaryn and Tomaszewska 2013, Tomaszewska and Szawaryn 2013). However, the research of Seago et al. (2011) includes only 7 species of the former subfamily Epilachninae, so the relationship between *Cynegetis* and other genera was not completely resolved. Ongoing research by W. Tomaszewska and K. Szawaryn on the phylogeny of Epilachninae and by A. Ślipiński et al. on the comprehensive molecular phylogeny of Coccinellidae may clarify generic relationships which will not be discussed here.

The species of *Cynegetis* are very similar to *Subcoccinella* Agassiz & Erichson in having oval and strongly convex bodies, well developed spurs on all tibiae and similar male and female genitalia. Kapur (1950) described larvae of these two genera, which also supports their close relationships based on the similar general shape and the armature of the body wall.

*Cynegetis* is a very small genus, containing only two species: *Cynegetis impunctata* (Linnaeus, 1958) and *Cynegetis syriaca* (Mader, 1958), which are distributed in the Palaearctic region (Jadwiszczak and Węgrzynowicz 2003). This genus was unknown from China until a new species, described in the present paper, was found during comprehensive investigations of Chinese ladybirds by the members of Shunxiang Ren’s research group.

## Material and methods

The external morphology was observed with a dissecting stereoscope (SteREO Discovery V20, Zeiss and Leica Mz Apo). The following measurements were made with an ocular micrometer: total length, length from apical margin of clypeus to apex of elytra (TL); total width, width across both elytra at widest part (TW = EW); height, from the highest part of the beetle to elytral outer margins (TH); head width in a frontal view, widest part including eyes (HW); pronotal length, from the middle of anterior margin to the base of pronotum (PL); pronotal width at widest part (PW); elytral length, along the suture, from the apex to the base including the scutellum (EL). Male and female genitalia were dissected, cleared in a 10% solution of NaOH by boiling for several minutes, and examined with an Olympus BX51 and Leica compound microscopes.

Morphological character photographs were made with digital cameras (AxioCam HRc and Coolsnap–Pro*cf* & CRI Micro*Color), connected to the dissecting microscope. The software AxioVision Rel. 4.8 and Image-Pro Plus 5.1 were used to capture images from both cameras, and photos were cleaned up and laid out in figs with Adobe Photoshop 8.0 CS.

Morphological terms of Coccinellidae follow Ślipiński (2007) and Ślipiński and Tomaszewska (2010). Type specimens designated in the present paper are deposited at SCAU – the Department of Entomology, South China Agriculture University, Guangzhou, China. Specimens of *Cynegetis impunctata* and *Subcoccinella vigintiquatuorpunctata* (L.) examined for comparison are deposited at: ANIC – Australian National Insect Collection, CSIRO, Canberra, Australia, BPBM – Bernice P. Bishop Museum, Honolulu, USA; IOZ – the Institute of Zoology, Chinese Academy of Sciences, Beijing, China.

## Taxonomy

### Genus Cynegetis

Taxon classificationAnimaliaColeopteraCoccinellidae

Chevrolat

Cynegetis Chevrolat in Dejean 1837: 461. Type species: *Coccinella impunctata* Linnaeus, 1767, subsequent designation by Crotch (1874).
Cycnegetis (sic!): Crotch 1874: 90.

#### Diagnosis.

**Figure 1.**
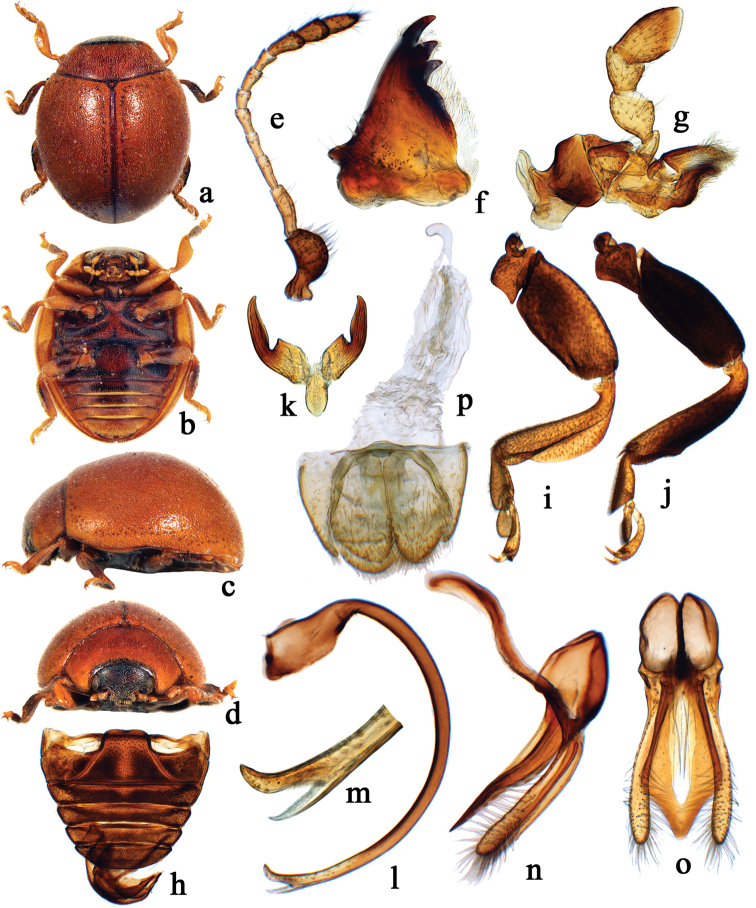
Morphological characters of the genus *Cynegetis*. **a–o** *Cynegetis impunctata* Linnaeus, 1767 From Poland. **a** dorsal habitus **b** ventral habitus **c** lateral habitus **d** frontal habitus **e** antenna **f** mandible **g** maxilla **h** abdomen **i** front leg **j** hind leg **k** tarsal claw **l–o** male genitalia: **l** penis **m** apex of penis **n** tegmen, lateral view **o** tegmen, ventral view **p** female genitalia: coxites and spermatheca.

**Figure 2.**
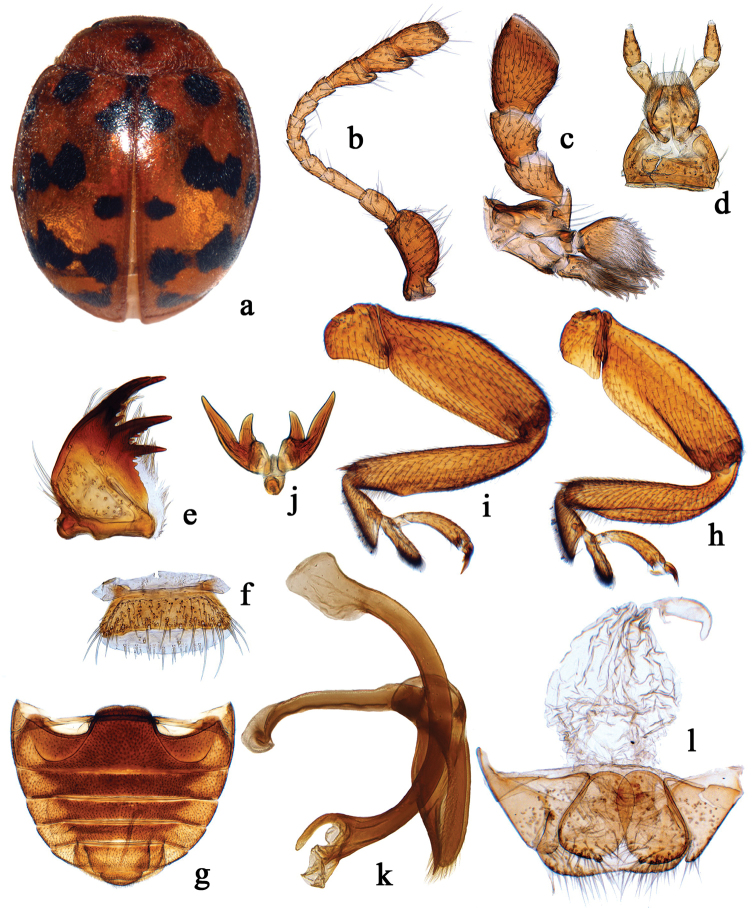
Morphological characters of the genus *Subcoccinella*. **a–l** *Subcoccinella vigintiquatuorpunctata* Linnaeus, 1758 From China. **a** dorsal habitus; **b** antenna **c** maxilla **d** labium **e** mandible **f** labrum **g** abdomen **h** front leg **i** hind leg **j** tarsal claw **k** male genitalia: penis and tegmen **l** female genitalia: coxites and spermatheca.

**Figure 3.**
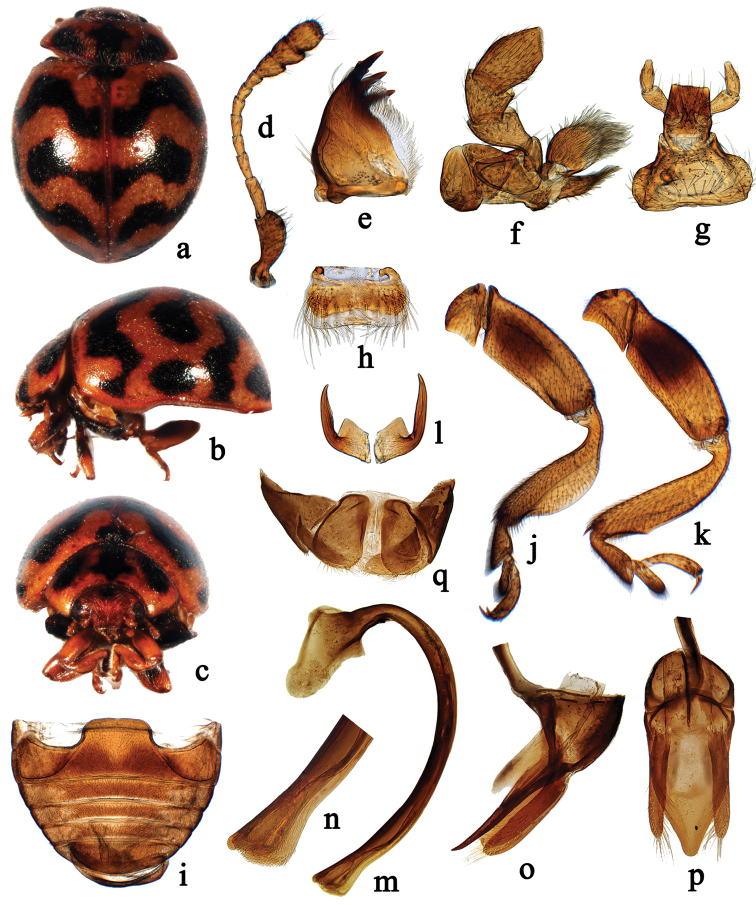
*Cynegetis chinensis* Wang & Ren, sp. n. **a** dorsal habitus **b** lateral habitus **c** frontal habitus **d** antenna **e** mandible **f** maxilla **g** labium **h** labrum **i** abdomen **j** front leg **k** hind leg **l** tarsal claw **m–p** male genitalia: **m** penis **n** apex of penis **o** tegmen, lateral view **p** tegmen, ventral view **q** female genitalia: coxites.

This genus is most similar to *Subcoccinella* in general shape of the body and the genitalia of both sexes and sharing interocular distance of more than 0.75 width of head (Figs 1a, 1l–p, 2a, 2k, 2l, 3a, 3m–q). *Cynegetis*, however, can be distinguished from *Subcoccinella* by the strongly convex body, anterior margin of clypeus distinctly emarginate, subapical teeth and incisor edge of mandible without denticles (Figs 1f, 3e), the terminal maxillary papomere barrel shaped (Figs 1g, 3f), short metaventrite (Fig. 1b), elytral surface covered with double-sized punctures, elytral epipleuron with distinct foveae for apices of mid and hind femora, strongly expanded/inflated outer edges of front tibiae (Figs 1i, 3j), and tarsal claw single, possessing large basal tooth (Fig. 1k, l). In *Subcoccinella*, the body is moderately convex, anterior margin of clypeus is straight or weakly emarginate, subapical teeth and incisor edge of mandibles are multidentate (Fig. 2e), the terminal maxillary palpomere is elongate and widened apically (Fig. 2c), the metaventrite is relatively long, the elytral surface covered with single-sized punctures, the elytral epipleuron smooth without foveae, outer edges of tibiae of front legs simple (Fig. 2h), and tarsal claws are bifid, lacking basal tooth (Fig. 2j).

#### Description.

Body shortened oval, strongly convex, dorsum densely pubescent (Figs 1a–d, 3a–c). Head with frons slightly convex. Clypeus protruded with anterior margin distinctly emarginate at middle. Labrum transverse, covered with densely distributed, long setae, anterior margin emarginate. Mandible subtriangular in shape, with two apical teeth and two subapical teeth: apical teeth long and large with 4–5 additional denticles; subapical teeth shorter than apical ones, smooth without additional denticles (Figs 1f, 3e). Antennae with 11 antennomeres (Figs 1e, 3d), antennal insertions located posterior to imaginary line joining anterior edges of eyes (Figs 1d, 3c). Maxillary palp with terminal palpomere longer than wide, barrel shaped, truncate at apex (Figs 1g, 3f); lacinia hook-like or simple. Terminal labial palpomere elongate, narrowing anteriorly (Fig. 3g).

Pronotum transverse, widest at base and gradually narrowing anteriorly, anterior and hind margins not bordered, anterior angles distinctly protruded. Scutellum small, triangular. Elytra at base distinctly wider than pronotum, lateral margins very narrow, invisible from above, humeral angles inconspicuous. Prothoracic hypomeron with clearly delimited cavities to accommodate apices of femora of front legs. Prosternum T-shaped, without carinae (Fig. 1b).

Mesoventrite with anterior edge with complete raised border and with weak groove behind it, mesal surface with cavity for receiving prosternal process; meso-metaventral junction broad, forming an almost straight line or slightly emarginated. Metaventrite about as long as abdominal ventrite 1 with incomplete discrimen (Fig. 1b); metaventral postcoxal lines recurved, complete laterally. Elytral surface with double-sized punctures; epipleuron incomplete apically, with distinct foveae for apices of femora of mid and hind legs (Fig. 1b).

Fore and mid trochanters angulate, produced. Fore tibia with single apical spur; mid and hind tibiae with two spurs (Figs 1i–j, 3j–k). Mid and hind tibiae on outer edge near apex with oblique carina. Tarsal claws simple with subquadrate tooth at base (Figs 1k, 3l).

Abdomen with six ventrites in males and five ventrites in females; abdominal postcoxal lines recurved roundly, almost complete (Figs 1h, 3i).

Male genitalia. Tegmen stout, penis guide wide and flat in ventral view, parameres straight with densely distributed setae apically (Figs 1n–o, 3o–p). Penis stout, curved; basal capsule expanded, but not typically T-shaped; apex bifid or not (Figs 1l–m, 3m–n).

Female genitalia. Coxites oval, setose apically; styli present or absent. (Figs 1p, 3q). Spermatheca small, curved weakly sclerotized (Fig. 1p).

#### Distribution.

China: Ningxia; Europe (Austria, Belgium, Bosnia and Herzegovina, Czech Republic, Denmark, Finland, France, Germany, Hungary, Italy, Norway, Poland, Romania, Slovakia, Spain, Sweden, Switzerland, West Russia); Asia: Asian part of Russia (Maritime Prov.), Iran, N. Korea, Syria, Turkey.

#### Key to the species of *Cynegetis*

**Table:** 

1	Antennomere 7 subquadrate; elytra covered with black, transverse, irregularly shaped bands (Fig. 3a–b)	***Cynegetis chinensis* Wang & Ren, sp. n.**
–	Antennomere 7 distinctly elongate; elytra with separated black spots or without black spots	**2**
2	Maxillary lacinia strongly sclerotized, hook-like; head always black; apex of penis bifid (Fig. 1l–m); the length of parameres almost equal to penis guide	***Cynegetis impunctata* (Linnaeus)**
–	Maxillary lacinia moderately sclerotized, simple; head often brown; elytra yellowish brown with black spots; apex of penis not bifid; the length of parameres distinctly shorter than penis guide	***Cynegetis syriaca* (Mader)**

### Cynegetis chinensis

Taxon classificationAnimaliaColeopteraCoccinellidae

Wang & Ren sp. n.

http://zoobank.org/641DABF3-E7AB-4263-8475-32750EC18055

Figure 3

#### Diagnosis.

This species is very similar to *Cynegetis syriaca* in general appearance and male genitalia but it can be distinguished from the latter as follow: antennomere 7 subquadrate, scutellum black, most of black spots on elytra joined, forming wavy shaped bands, coxites bearing distinct styli and the characters of penis capsule and penis guide are distinctly different from the latter. In *Cynegetis syriaca*, antennomere 7 is distinctly elongate, scutellum yellow, black spots on the elytra are separated from each other and the coxites lack styli (Fürsch 1986, Duverger 1983, WT, personal observations).

#### Description.

TL: 3.4–3.5 mm, TW: 2.7–2.9 mm, TH: 1.7–1.9 mm, TL/TW: 1.21–1.26; PL/PW: 0.44–0.45; EL/EW: 1.00–1.03; HW/TW: 0.38; PW/TW: 0.67.

Body short oval, dorsum strongly convex, densely pubescent (Fig. 3a–c). Head yellowish brown, with small black spot at base. Pronotum yellowish brown, with three large black spots, the middle one longitudinal, expanded laterally at apical 1/4, the lateral pair irregularly oval. Scutellum black. Elytra yellowish brown, with three rounded black spots and three black wavy bands, arranged as Fig. 3a–b. Underside yellowish brown, meso- and metaventrite black, epipleura and legs yellow.

Head with frontal punctures fine and inconspicuous, associated with scattered long setae; eyes of small size and moderately coarsely faceted. Maxillary lacinia moderately sclerotized, simple. Pronotal disk with fine and densely distributed punctures, slightly larger than those on head, 0.5–1.0 diameters apart. Elytral disk with punctures similar to those on pronotum. Prosternum and mesoventrite rough, with scattered short setae. Metaventrite broad with fine and inconspicuous punctures.

Male genitalia. Penis stout, strongly curved, apex slightly expanded, truncate with scattered short setae, basal capsule large (Fig. 3m–n). Tegmen stout and symmetrical (Fig. 3o–p). Penis guide in lateral view widest at base and narrowing to pointed apex, the basal 1/2 with a membrane part which accept stout penis (Fig. 3o). Parameres rather narrow and almost straight, distinctly shorter than penis guide (Fig. 3o). Penis guide in ventral view flattened, widest at basal 1/3, strongly narrowing to apex, apex blunt (Fig. 3p).

Female genitalia. Coxites oval, with distinct terminal styli (Fig. 3q). Spermatheca not studied.

#### Types.

**Holotype:** 1 male, **China, Ningxia:** Baiyunsi, Liupanshan National Natural Reserve, Jingyuan County, 106°15.6'E, 35°36.6'N, ca2300m, 10.viii.2009, Wang XM leg; **Paratypes:** 1 female, same data as holotype.

#### Distribution.

China (Ningxia).

#### Etymology.

The specific epithet is an adjective derived from the geographical name “China”, the type locality of this ladybird.

## Supplementary Material

XML Treatment for Cynegetis

XML Treatment for Cynegetis chinensis
